# Editorial: Women in pediatric oncology Vol II: 2022

**DOI:** 10.3389/fonc.2023.1223296

**Published:** 2023-06-26

**Authors:** Yong-mi Kim, Joanna B. Kitlinska

**Affiliations:** ^1^ Children’s Hospital Los Angeles, Department of Pediatrics, Keck School of Medicine, University of Southern California, Los Angeles, CA, United States; ^2^ Biochemistry and Molecular & Cellular Biology, Georgetown University, Washington, DC, United States

**Keywords:** pediatric oncology, neuroblastoma, survivorship, rhabdoid tumor, angiosarcoma, ovarian mass, pediatric-type diffuse gliomas, myeloid sarcoma

120 years ago, in December 1903, Marie Curie became the first woman to win a Nobel Prize. Since then, women have made numerous contributions to all fields of science. Yet, despite this undisputable progress, female scientists still remain a minority. Hence, it is particularly important to highlight and promote their work. This edition of the Research Topic is devoted to women involved in childhood cancer research.

While pediatric cancer research has led to advances in our understanding of the biology of childhood cancers, and thereby contributed to advances in diagnosis, treatment and survivor psychosocial care, there remains an unmet need for improving patient outcomes and quality of life of the childhood cancer survivors. The second volume of “*Women in Pediatric Oncology*” contains 15 articles spanning a broad range of topics, starting from basic science through clinical research and survivorship care. Sorteberg et al. identify the activation of cyclin dependent kinase p21^Cip/Waf1^ as a potential mechanism of chemoresistance in high-risk neuroblastoma and present preclinical data demonstrating efficacy of its inhibitor in combination with routine chemotherapeutics. Cervi et al. report a complete response to Trk inhibitor treatment in a patient with angiosarcoma carrying KHDRBS1-NTRK3 fusion gene, the first such case and a perfect example of precision-based medicine. A review paper by Cruz-Galvez et al. provides a comprehensive overview of retinoblastoma – the known facts and novel findings pertaining to this classic, genetically-driven pediatric malignancy.

Women are also pioneering novel technologies and treatment approaches. Two papers by Petrilli et al. and Miller et al. describe the use of state-of-the-art molecular profiling methods to characterize heterogeneity of rhabdoid tumors and pediatric-type diffuse high-grade gliomas, respectively. Other articles in this collection focus on clinical practice and outcomes research. Samborska et al. describe treatment outcomes in patients with myeloid sarcoma, while Puglisi et al. focus on the clinical characteristics of patients with combined neuroblastic tumors and neurofibromatosis type 1. Ariagno et al. present a timely study on the impact of prior COVID-19 infection on the risk of endothelial dysfunction in pediatric and adolescent patients undergoing hematopoietic cell transplants.

The clinical practice in pediatric oncology has to be tailored to children and often does not follow the same protocols as the care for adult patients. This problem is emphasized by Wang et al., who evaluate a diagnostic performance of imaging techniques in children with ovarian masses. On the other hand, Reschke et al. describe the development of clinical protocols aiming at improving multidisciplinary care of children with high-risk malignancies.

The last group of the manuscripts included in this Research Topic focuses on psychosocial issues associated with care for pediatric and adolescent cancer patients. These studies range from challenges in communication between health providers and patients and/or their families, as well as everyday difficulties facing the patients, their caregivers and educators (Burgers et al.; McLoone et al.; Otth et al.; Otth and Scheinemann; Rockwell et al.).

Altogether, this collection of outstanding 15 articles is a perfect example of the scope and variety of research performed by women scientists focusing on pediatric oncology and hematology. After reading the collection of these articles, the reader will appreciate the multi-faceted approach of current childhood cancer research, which remains a work in progress.

## Author contributions

JK and Y-MK reviewed and summarized the manuscripts published in the Research Topic. All authors contributed to the article and approved the submitted version.

